# *Bombyx mori β-1,3-Glucan Recognition Protein 4* (*BmβGRP4*) Could Inhibit the Proliferation of *B. mori* Nucleopolyhedrovirus through Promoting Apoptosis

**DOI:** 10.3390/insects12080743

**Published:** 2021-08-18

**Authors:** Jie Wang, Lin-Bao Zhu, Yan Ma, Ying-Xue Liu, Hui-Hua Cao, Yu-Ling Wang, Xue Kong, Zhi-Hao Huang, Han-Dan Zhu, Yan-Xiang Wang, Shi-Huo Liu, Jia-Ping Xu

**Affiliations:** 1 School of Life Sciences, Anhui Agricultural University, Hefei 230036, China; wangjie_3001@163.com (J.W.); zhulinbao@163.com (L.-B.Z.); matafeiyan2016@163.com (Y.M.); liuyingxue688@163.com (Y.-X.L.); chh18856960204@163.com (H.-H.C.); 15755072270@163.com (Y.-L.W.); kx18895706038@163.com (X.K.); hzh879546213@163.com (Z.-H.H.); zhdwangyiyouxiang@163.com (H.-D.Z.); wxzgdx@163.com (Y.-X.W.); 2 Anhui International Joint Research and Developmental Center of Sericulture Resources Utilization, Hefei 230036, China

**Keywords:** *Bombyx mori*, β-glucan recognition protein, *B. mori* nucleopolyhedrovirus, apoptosis

## Abstract

**Simple Summary:**

In this study, a differentially expressed *β-1,3-glucan recognition protein*, *BmβGRP4*, was identified from a transcriptome database. Significant downregulation of *BmβGRP4* expression was detected after BmNPV infection in the P50 larvae midgut. Subsequently, the overexpression of *BmβGRP4* suppressed BmNPV proliferation in BmN cells; however, the siRNA-mediated knockdown of *BmβGRP4* facilitated BmNPV proliferation in *B. mori* larvae. Furthermore, we demonstrated that *BmβGRP4* overexpression promoted BmNPV induced cellular apoptosis. Then, we found that *BmβGRP4* positively regulated *BmPTEN* and negatively regulated *BmIAP*. Consequently, we speculated that BmNPV inhibited *BmβGRP4* to suppress *BmPTEN* and facilitate *BmIAP* to inhibit cell apoptosis to evade host antiviral defense. These findings will lay a foundation for further study of the functions of *BmβGRP4* in response to BmNPV.

**Abstract:**

β-1,3-glucan recognition proteins (βGRPs) as pattern recognition receptors (PRRs) play an important role in recognizing various pathogens and trigger complicated signaling pathways in insects. In this study, we identified a *Bombyx mori* β-1,3-glucan recognition protein gene named *BmβGRP4*, which showed differential expression, from a previous transcriptome database. The full-length cDNA sequence was 1244 bp, containing an open reading frame (ORF) of 1128 bp encoding 375 amino acids. *BmβGRP4* was strongly expressed in the larval stages and highly expressed in the midgut of *B. mori* larvae in particular. After BmNPV infection, the expression of *BmβGRP4* was reduced significantly in the midgut. Furthermore, a significant increase in the copy number of BmNPV was observed after the knockdown of *BmβGRP4* in 5th instar larvae, while the overexpression of *BmβGRP4* suppressed the proliferation of BmNPV in BmN cells. Subsequently, the expression analysis of several apoptosis-related genes and observation of the apoptosis morphology demonstrated that overexpression of *BmβGRP4* facilitated apoptosis induced by BmNPV in BmN cells. Moreover, *BmβGRP4* positively regulated the phosphatase and tensin homolog gene (*BmPTEN*), while expression of the inhibitor of apoptosis gene (*BmIAP*) was negatively regulated by BmβGRP4. Hence, we hypothesize that BmNPV infection might suppress *BmPTEN* and facilitate *BmIAP* to inhibit cell apoptosis by downregulating the expression of *BmβGRP4* to escape host antiviral defense. Taken together, these results show that *BmβGRP4* may play a role in *B. mori* response to BmNPV infection and lay a foundation for studying its functions.

## 1. Introduction

The silkworm *Bombyx mori* is a holometabolism lepidopteran insect, which has been domesticated for about 5700 years from the wild progenitor *B. mandarina* [1]. It is used as a model organism in insect genetics and immunology fundamental research [2,3]. *B. mori* nucleopolyhedrovirus (BmNPV) is a serious viral pathogen that specifically infects the domestic silkworm, causing severe economic loss in sericulture around the world [4]. In recent years, many genes and proteins involved in BmNPV infection have been identified by different approaches, such as serine protease-2, lipase-1, alkaline trypsin protein and NADPH oxidoreductase (BmNox) [5]. Furthermore, these genes are expressed differentially in the resistant strains compared to the susceptible strain, and the results confirm the correlation of these genes to BmNPV resistance in *B. mori* [6]. Previous studies attempted to explore this resistance mechanism mainly using transcriptomic or proteomic analysis [7,8]. Xue et al. [9] found that numerous differentially expressed genes were mainly involved in the cytoskeleton, transcription, translation and energy metabolism in Bm5 cells after BmNPV infection based on transcriptome analysis. Dong et al. [10] identified a receptor expression-enhancing protein (REEP) using iTRAQ-based quantitative protein expression profiling, indicating that BmREEPa was required for BmNPV infection. However, the interaction of β-1,3-glucan recognition proteins (βGRPs) with BmNPV has not yet been reported. 

The innate immune system is an evolutionally conserved mechanism that can protect the host from invading pathogens including viruses [11]. In invertebrates, this system is activated in the presence of cell wall components from microbes like lipopolysaccharide (LPS), β-1,3 glucan (βG) and peptidoglycan (PG), together known as pathogen-associated molecular patterns (PAMPs) [12]. PAMPs are recognized by pattern recognition receptors (PRRs) in cells of the innate immune system, which can be divided into membrane-bound PRRs, receptor kinases and the mannose receptor [13]. PRRs have been identified in many insect species, including *Drosophila melanogaster* and *Manduca sexta* [14,15]. The most important PRRs purified from invertebrates include β-1,3 glucan binding protein (β-GBP), lipopolysaccharide and β-1,3 glucan binding protein (LGBP), Gram negative bacteria binding protein (G-NBP), peptidoglycan recognition protein (PGRP) and C-type lectin [16]. PGRPs and βGRPs are pattern recognition molecules that can recognize peptidoglycans in the bacterial cell wall and β-1,3-glucan from fungi, respectively, which play an important role in innate immunity activation and regulation [17,18]. The recognition of invading pathogen cells will trigger complex signaling pathways that eventually lead to the synthesis of effector molecules such as cytokines and antimicrobial peptides. Extracellular serine proteinase systems have evolved in invertebrates to mediate these processes [19]. One of these proteinase cascades causes proteolytic activation of prophenoloxidase (proPO), and active phenoloxidase (PO) generates quinones that are intermediates for melanization [20]. 

To date, β-1,3-glucan recognition proteins have been identified from many invertebrate groups. Ochiai et al. [21] were the first to purify these proteins from *B. mori*. Huang et al. [22] cloned a β-1,3-glucan recognition protein from *P. xylostella*, and suggested that it played a vital role in response to the expression of antimicrobial peptide (AMP) genes. Additionally, a 52 kDa β-1,3-glucan recognition protein isolated from *M. sexta* was shown to aggregate bacteria and fungi to stimulate PPO activation [17]. All of these results indicate that βGRPs could be involved in insect innate immune response against pathogens. The innate immune response to a virus is critical for mobilizing protective immunity. Host cells of the innate immune system can utilize PRRs to identify viral pathogens by engaging PAMPs [23]. The antiviral innate immune response is induced depending on the recognition of viral components by host PRRs [24]. Heiberg et al. [25] revealed a toll-like receptor (TLR)-mediated inflammatory response in children with chronic hepatitis B (CHB) virus infection. Tsai et al. [26] found that the expression of PGRP-SB1 and PGRP-SD was upregulated after Sigma virus infection. Gao et al. [27] also revealed that BmPGRP-S3 played an important role in the immune response of silkworms to *B. mori* cytoplasmic polyhedrosis virus (BmCPV). However, the function of βGRPs in virus infection is still poorly understood, especially for BmNPV.

In this study, we identified a pattern recognition receptor *BmβGRP4* from a *B. mori* transcriptome database and investigated its role in BmNPV infection. The spatiotemporal expression pattern of *BmβGRP4* was analyzed and a significant downregulation was detected after BmNPV infection in the midgut. Subsequently, overexpression and siRNA-mediated knockdown of *BmβGRP4* were used to explore its influence on BmNPV in BmN cells and *B. mori* larvae. Furthermore, we demonstrated that *BmβGRP4* promoted BmNPV-induced cellular apoptosis. Then, we found that BmβGRP4 positively regulated *BmPTEN* and negatively regulated *BmIAP*. Consequently, we speculated that BmNPV inhibited *BmβGRP4* to suppress *BmPTEN* and facilitated *BmIAP* to inhibit cell apoptosis to evade host antiviral defense. These results would lay the foundation for further study of the functions of *BmβGRP4* in response to BmNPV.

## 2. Materials and Methods

### 2.1. Silkworm Larvae, Cell Line and Virus Preparation

The silkworm P50 strain was maintained in the Key Laboratory of Sericulture, Anhui Agricultural University, Hefei, China. The larvae were fed using fresh mulberry leaves. The first three instar larvae were reared at 26 ± 1 °C, 75 ± 5% relative humidity with 12 h day/night cycles, and the last two instar larvae were reared at 24 ± 1 °C, with the same relative humidity and photoperiod as above. The BmN cell line was cultured in TC-100 medium (United States Biological, Swampscott, MA, USA) supplemented with 10% (volume ratio, *v*/*v*) fetal bovine serum, 100 μg mL^−1^ penicillin and 30 μg mL^−1^ streptomycin (Gibco, Rockville, MD, USA) at 27 °C.

The BmNPV T3 strain was kept in our laboratory. On the 1st day of the 5th instar, all larvae were starved for 24 h, then administered 5 μL BmNPV suspended in water (1.0 × 10^6^ OBs mL^−1^) *per os*, and the control was treated with 5 μL sterile water. Larvae midguts were collected at different time points post infection.

Budded virus of BmNPV containing an EGFP tag (BV-EGFP) was generously donated by Doctor Xue-Yang Wang from Jiangsu University of Science & Technology and was kept in our laboratory. The culture containing BV-EGFP (1.0 × 10^8^ pfu mL^−1^) was used to infect BmN cells in this study.

### 2.2. Identification and Phylogenetic Analysis of BmGRP4

In the previous study, a comparative transcriptome analysis was employed to identify differential expressed genes (DEGs) in response to BmNPV infection [7]. In this study, *BmβGRP4* was identified from the dataset through the National Center for Biotechnology Information non-redundant (NCBI-nr) database using a basic local alignment search tool (BLAST). The presence and location of the signal peptide was predicted by the SignalP-5.0 Server (http://www.cbs.dtu.dk/services/SignalP) accessed on 5 April 2021. The molecular weight (MW) and isoelectric point (pI) of the BmβGRP4 protein were calculated using ExPASy software (http://web.expasy.org/compute_pi/). Conserved domains were determined using the NCBI conserved domain database (http://www.ncbi.nlm.nih.gov/Structure/cdd/wrpsb.cgi) and the SMART online software (http://smart.embl-heidelberg.de/). The phylogenetic tree was constructed with MEGA X software using the neighbor-joining method with 1000-fold bootstrap resampling [28].

### 2.3. RNA Isolation, cDNA Synthesis and RT-qPCR Analysis

Total RNA was extracted from different samples using TRIzol reagent (Invitrogen, Grand Island, NY, USA) according to the manufacturer’s instructions. The first-strand cDNA was synthesized using a PrimeScript^TM^ RT kit with gDNA Eraser (TaKaRa, Dalian, China) according to the manufacturer’s instructions. Quantitative reverse transcription PCR (RT-qPCR) analysis was performed using the CFX96 real-time system (Bio-Rad, Singapore) with a SYBR Premix ExTaq^TM^ Kit (TaKaRa), as described previously [29]. The primers used in the RT-qPCR are shown in Table S2. The 2^−ΔΔCt^ method was adopted to calculate the relative expression levels. In this study, *B. mori glyceraldehyde-3-phosphate dehydrogenase* (*BmGAPDH*) was used as the reference gene. The statistical significance between treatments was analyzed using SPSS Statistics software (V 26.0. IBM, NY, USA). Three biological replicates were used.

### 2.4. Prokaryotic Expression and Protein Purification

In order to analyze the function of *BmβGRP4* at the protein level, we expressed recombinant BmβGRP4 protein using a prokaryotic expression system. The primers are listed in Table S2 (the underlined portions indicate the restriction enzyme sites). The DNA fragment contained mature *BmβGRP4* and was cloned and ligated into the expression vector pET-28a (Novagen, Madison, WI, USA) with restriction enzyme sites *EcoR I* and *Xho I*. The recombinant pET-28a-BmβGRP4 plasmid was confirmed by DNA sequencing and transformed into *Escherichia coli* BL21 competent cells (TransGen, Beijing, China). Recombinant BmβGRP4 protein expression was induced using 1 mM isopropyl-β-thiogalactopyranoside (IPTG) at 16 °C overnight. After centrifugation at 7500× *g* for 5 min at room temperature, the *E. coli* cells were suspended in phosphate-buffered saline (PBS, pH 7.4) and disturbed by sonication on ice. After centrifugation at 12,000× *g* for 20 min at 4 °C, the recombinant protein insoluble pellet was purified using High Affinity Ni-NTA Resin (GenScript, Nanjing, China), according to the manufacturer’s instructions. The quality of purified protein was analyzed by 12% sodium dodecyl sulfate-polyacrylamide gel electrophoresis (SDS-PAGE) followed by staining with Coomassie Brilliant Blue R250 and Western blot using anti-His primary antibody (1:5000, TransGen) and horseradish peroxidase (HRP)-conjugated goat anti-mouse secondary antibody (1:10,000, TransGen).

### 2.5. Antibody Preparation and Western Blot Analysis

The rabbit anti-BmβGRP4 and anti-VP39 antiserum were prepared by HuaAn Biotechnology Ltd (HUABIO, Hangzhou, China). The Western blot analysis was performed as described previously [8]. The primary antibodies, rabbit anti-BmβGRP4 antiserum (1:500) and mouse anti-β-Tubulin (1:1000, TransGen) as well as the secondary horseradish peroxidase (HRP)-conjugated goat anti-rabbit antibody (1:5000, TransGen) or anti-mouse antibody (1:5000, TransGen) were used in this study. The immobilized conjugates on the polyvinylidene fluoride (PVDF) membranes were visualized using an HRP-DAB Chromogenic Kit (TIANGEN, Beijing, China). Three biological replicates were used. 

### 2.6. RNAi by Injecting Small Interfering RNA in B. mori Larvae and BmNPV Infection

The small interfering RNA (siRNA) against *BmβGRP4* and siNC (negative control) were designed and synthesized by the GenePharma Biotech Company (GenePharma, Shanghai, China); the siRNA sequences are shown in Table S3. Thirty larvae on the 3rd day of the 5th instar were used for RNA interference experiments. Each larva was injected with 2 μg siRNA, and midgut tissues were obtained at 24 h and 48 h post injection. The RNAi efficiency was assessed using RT-qPCR and Western blot. 

Sixty larvae were injected with two kinds of siRNAs and divided into two groups (30 for siNC and 30 for siBmβGRP4). Then, all larvae were administered with 5 μL of BmNPV suspended in water (1 × 10^6^ OBs mL^−1^) *per os* at 24 h post injection of siRNA. The 10 larval midgut tissues in each group were obtained and pooled at 24, 48 and 72 h p.i. (hours post infection). Genomic DNA of the midgut was isolated using a Genomic DNA extraction kit (TaKaRa). The relative expression level of BmNPV *VP39* was measured by RT-qPCR to indicate the abundance of virus in infected larvae. The siNC injected larvae were used as controls in these experiments. Three biologically independent samples were used.

### 2.7. Overexpression of BmβGRP4 in BmN Cells and BmNPV Infection

The full-length sequence of *BmβGRP4* ORF was amplified from the cDNA of the *B. mori* midgut with OEBmβGRP4 primers (Table S2). The purified DNA fragment was ligated into a transient overexpression vector, pIZT-mCherry vector to construct an overexpression vector for pIZT-mCherry-BmβGRP4. The recombinant overexpression vector was confirmed by DNA sequencing, and transfected into BmN cells (1.0 × 10^6^ cells well^−1^) on Costar 6-well cell culture clusters using Neofect^TM^ DNA transfection reagent (NEOFECT, Beijing, China) according to the manufacturer’s instructions and cultured at 27 °C. The fluorescence signal was captured using an inverted microscope DMi8 camera (Leica, Solms, Germany), followed by culturing for 48 h after transfection. BmN cells were harvested for total RNA and protein extraction to assay the overexpression efficiency of *BmβGRP4* by RT-qPCR and Western blot. The pIZT-mCherry plasmid transfected BmN cells were used as a control.

The *BmβGRP4* overexpressed BmN cells (approximately 5 × 10^5^ cells well^−1^) were infected with 10 µL of culture medium containing BV-EGFP at a multiplicity of infection (MOI) of 2 in each well, and the pIZT-mCherry transfected cells were used as a control. The infected BmN cells were collected at 24, 48 and 72 h p.i. The change in BmNPV proliferation was determined by analyzing BmNPV *VP39* mRNA and protein levels through RT-qPCR and Western blot, as well as observing the green fluorescence using an inverted microscope DMi8 camera. Western blot analysis was performed as mentioned above. The primary antibodies, rabbit anti-VP39 antiserum (1:500, prepared by our laboratory), mouse anti-β-Tubulin (1:2000, TransGen) and the HRP-conjugated goat anti-rabbit secondary antibody or goat anti-mouse secondary antibody (1:5000, TransGen) were used in this study. Three biologically independent samples were used.

### 2.8. Detection of Apoptosis-Related Gene Expression and Observation of Apoptosis Morphology

The expression levels of apoptosis-related genes after BmNPV-induced apoptosis in *BmβGRP4* overexpressed BmN cells were detected by RT-qPCR analysis. The *BmβGRP4* overexpressed BmN cells were infected with BV-EGFP at an MOI of 2 and the cells were collected at 36 h p.i. to analyze the relative expression levels of *BmApaf1*, *BmDredd*, *BmBuffy*, *BmCaspaseNC*, *BmICE* and *BmCaspase1* through RT-qPCR; all of the primers are shown in Table S2. The pIZT-mCherry vector transfected cells were used as a control. Three biologically independent samples were used.

The apoptosis morphology of BmN cells was determined by fluorescence microscopy. First, BV-EGFP at an MOI of 2 was added to the *BmβGRP4* overexpressed BmN cells. Then, at 36 h p.i., all culture medium was removed, and the cells were fixed in 4% paraformaldehyde (PFA; Sangon) for 10 min at room temperature; after the removal of all 4% PFA, the cells were washed with PBS (pH 7.4) three times for 5 min each. After washing, the cell nuclei were stained with DAPI (Beyotime, Shanghai, China) in the dark for 10 min at room temperature. Finally, the cell morphology was examined using an inverted microscope DMi8 camera. The pIZT-mCherry vector transfected BmN cells were used as a control in this experiment. Three biologically independent samples were used.

### 2.9. Expression Level Analysis of BmPTEN and BmIAP

According to the demonstration of Jiang et al. [30], another important pattern recognition receptor BmPGRP2-2 was induced by BmNPV, through PTEN-phosphoinositide 3-kinase (PI3K)/Akt signaling to regulate apoptosis. Hence, we speculated that the pattern recognition receptor BmβGRP4 might also regulate apoptosis in response to BmNPV infection. In addition, Chen et al. [31] found that the *B. mori* inhibitor of apoptosis (*BmIAP*) gene had an inhibitory effect on apoptosis in silkworm cells. The expression levels of *BmPTEN* and *BmIAP* after *BmβGRP4* knockdown and overexpression were evaluated through RT-qPCR analysis using the specific primers of *BmPTEN* and *BmIAP* (Table S2).

## 3. Results

### 3.1. Identification of BmβGRP4 and Bioinformatics Analysis

**Figure 1:**
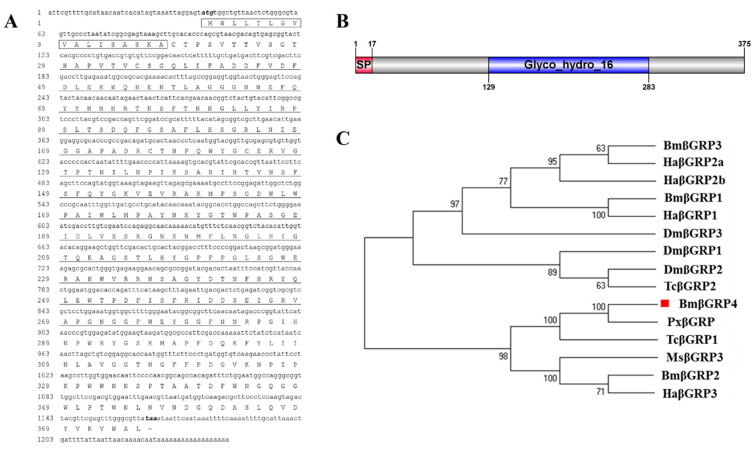
Bioinformatics analysis of *BmβGRP4* cDNA sequence. (**A**) Complete nucleotide sequence and deduced amino acid sequence of BmβGRP4. Numbers on the left side represent nucleotide and amino acid positions. The initiation codon (ATG) and termination codon (TAA) are indicated in black italics. The signal peptide is represented in the black box and the Glyco_hydro_16 domain is highlighted by a single line. (**B**) Structural domain of BmβGRP4 predicted using SMART software. The red box indicates the signal peptide and the blue box represents the Glyco_hydro_16 domain. (**C**) Phylogenetic relationships of BmβGRP4 in different species using the neighbor-joining method with a bootstrap value of 1000. The numbers at each node represent neighbor-joining distances. The red block indicates the BmβGRP4.

Based on the previous transcriptome database, a noteworthy DEG was filtered; we obtained the cDNA sequence of beta-1,3-glucan recognition protein 4 (βGRP4) precursor (Table S1). Based on NCBI BLAST results, the *BmβGRP4* (GenBank Accession No: NM_001166142.1) was identified. The cDNA sequence of BmβGRP4 contains an ORF of 1128 bp, which encodes 375 amino acids with a predicted MW of 42.0 kDa and an isoelectric point of 6.45. In addition, the first 17 amino acid residues (MWLLTLGVVALISASKA) at the N-terminus may act as a signal peptide for secretion (Figure 1A). Conserved domain prediction using SMART software suggested that the BmβGRP4 protein contained a Glyco_hydro_16 domain (129-283) (Figure 1B). In order to investigate the evolutionary relationships between BmβGRP4 and those of other insects, a phylogenetic tree was constructed by the neighbor-joining method (Figure 1C). The results show that BmβGRP4 had a high homology with βGRP from *P. xylostella*.

### 3.2. Tissue Distribution and Developmental Stage Expression of BmβGRP4

**Figure 2:**
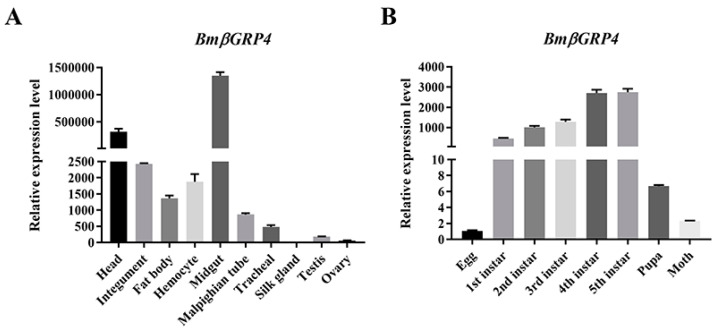
The spatiotemporal expression profiles of *BmβGRP4* in *B. mori* (P50 strain). The relative expression level of *BmβGRP4* in different tissues (**A**) and developmental stages (**B**) analyzed by RT-qPCR. Data were normalized using *BmGAPDH* and are represented as means ± SEM from three independent experiments. The relative expression level was calculated using the 2^−ΔΔCt^ method.

To explore the expression profile of *BmβGRP4* in different tissues, including the head, integument, midgut, hemocyte, fat body, malpighian tube, testis and ovary, the expression level of *BmβGRP4* was analyzed by RT-qPCR. The results indicated that *BmβGRP4* had high expression in the head, integument, hemocyte, fat body and midgut (Figure 2A). An analysis of the developmental stages revealed that *BmβGRP4* was expressed higher in the larval stage compared to the adult, and the expression increased gradually at each instar (Figure 2B).

### 3.3. Recombinant BmβGRP4 Expression, Purification and Antiserum Preparation

**Figure 3:**
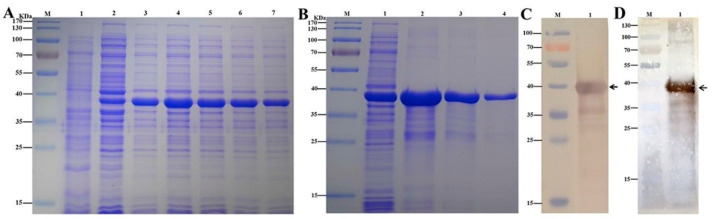
(**A**) Analysis of recombinant BmβGRP4 by SDS-PAGE. M: molecular protein markers. Lane 1: blank control without insert. Lane 2: negative control without induction. Lanes 3–7: induced expression with 0.2, 0.4, 0.6, 0.8, 1.0 mM final concentrations of IPTG, respectively. (**B**) SDS-PAGE analysis of purified recombinant proteins by affinity chromatography. M: molecular protein markers. Lane 1: recombinant protein without purification. Lanes 2–4: purified recombinant BmβGRP4 protein. (**C**) Western blot analysis of recombinant His-tagged BmβGRP4 protein identified by anti-His antibody. (**D**) Western blot analysis of recombinant BmβGRP4 identified by anti-BmβGRP4 antiserum. M: molecular protein markers. Lane 1: recombinant BmβGRP4.

To further analyze the function of *BmβGRP4*, recombinant His-tagged BmβGRP4 was expressed in *E. coli*. The recombinant BmβGRP4 with an MW of approximately 40 kDa was detected by 12% SDS-PAGE (Figure 3A). The recombinant protein was purified (Figure 3B) and used for antiserum preparation. The correct expression was confirmed by Western blot using the anti-His primary antibody and goat anti-mouse secondary antibody (Figure 3C). The antiserum was confirmed to detect the protein level of BmβGRP4 by Western blot (Figure 3D).

### 3.4. Expression Pattern Analysis of BmβGRP4 following BmNPV Infection

**Figure 4:**
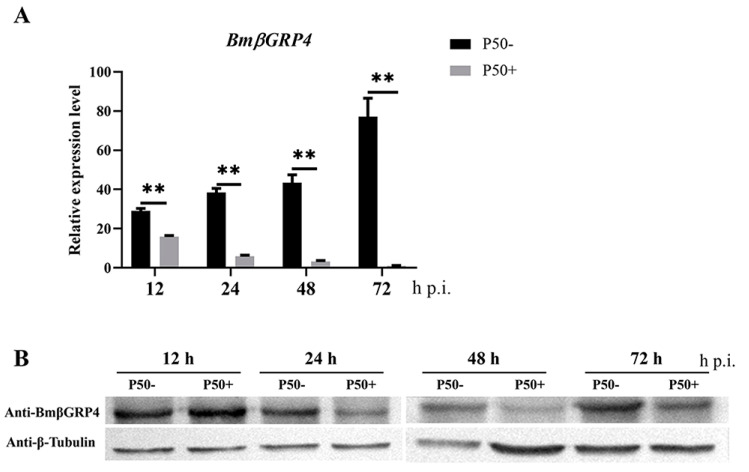
The expression levels of the *BmβGRP4* gene in the midgut of P50 strain larvae post BmNPV infection. The relative expression levels of *BmβGRP4* analyzed by RT-qPCR (**A**) and the translational levels of BmβGRP4 analyzed by Western blot (**B**) in the midgut at 12, 24, 48 and 72 h p.i. The relative expression level was calculated using the 2^−ΔΔCt^ method. Data were normalized using *BmGAPDH* and are represented as means ± SEM from three independent experiments. Statistical analysis was conducted using SPSS software with Student’s *t*-test. Significant differences are indicated by ** (*p* < 0.01). In the Western blot analysis, BmβGRP4 was detected using a rabbit anti-BmβGRP4 antiserum and β-Tubulin was used as an internal reference. “P50-” indicates non-infection and “P50+” indicates BmNPV-infection. h p.i., hours post infection.

Tissue expression patterns showed that *BmβGRP4* had the highest expression in the midgut. In order to investigate the possible role of *BmβGRP4* in response to BmNPV infection, RT-qPCR and Western blot were performed to determine BmβGRP4 expression patterns in the midgut at different time points post infection. The results suggest that the expression level of *BmβGRP4* was significantly downregulated from 12 to 72 h p.i. in the midgut compared with the control group (Figure 4A). Additionally, the results of Western blot analysis show that the protein levels of BmβGRP4 were also downregulated in the midgut from 24 to 72 h p.i. (Figure 4B). Based on the above results, we speculated that *BmβGRP4* could respond to BmNPV infection in the *B. mori* midgut.

### 3.5. Knockdown of BmβGRP4 by RNAi Facilitated Virus Proliferation in B. mori Larvae

**Figure 5:**
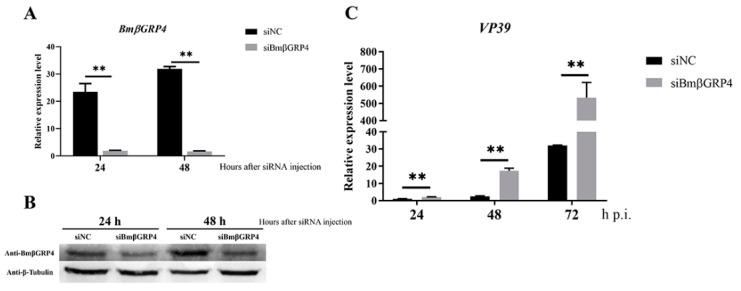
Knockdown of *BmβGRP4* promoted the proliferation of BmNPV in P50 strain larvae midgut. The interference efficiency of siBmβGRP4 was confirmed by analyzing the transcript (**A**) and translational (**B**) levels at 24 and 48 h after siRNA injection using RT-qPCR and Western blot. (**C**) BmNPV load change upon BmNPV infected in knockdown of BmβGRP4 larvae was determined by estimating the relative expression level of BmNPV *VP39* by RT-qPCR. Data were normalized using *BmGAPDH* and are presented as means ± SEM from three independent experiments. Statistical analysis was conducted using SPSS software. Significant differences are indicated by ** (*p* < 0.01).

To further determine whether *BmβGRP4* was related to BmNPV resistance in vivo, we examined the proliferation of BmNPV in *B. mori* larvae in which *BmβGRP4* was depleted. The knockdown of *BmβGRP4* was confirmed through analyzing transcriptional levels by RT-qPCR and translational levels by Western blot; effective for interfering with the expression of *BmβGRP4* in the midgut were observed at both 24 and 48 h post siRNA injection (Figure 5A,B). *B. mori* larvae were injected with siBmβGRP4 or siNC for 24 h prior to infection with BmNPV *per os*; compared with the control, viral loads represented by the relative expression level of *VP39* were remarkably increased from 24 to 72 h p.i. (Figure 5C). Hence, we concluded that knocking down *BmβGRP4* expression by RNAi in *B. mori* larvae could promote BmNPV proliferation in the midgut.

### 3.6. Overexpression of BmβGRP4 Suppressed Virus Proliferation in BmN Cells

**Figure 6:**
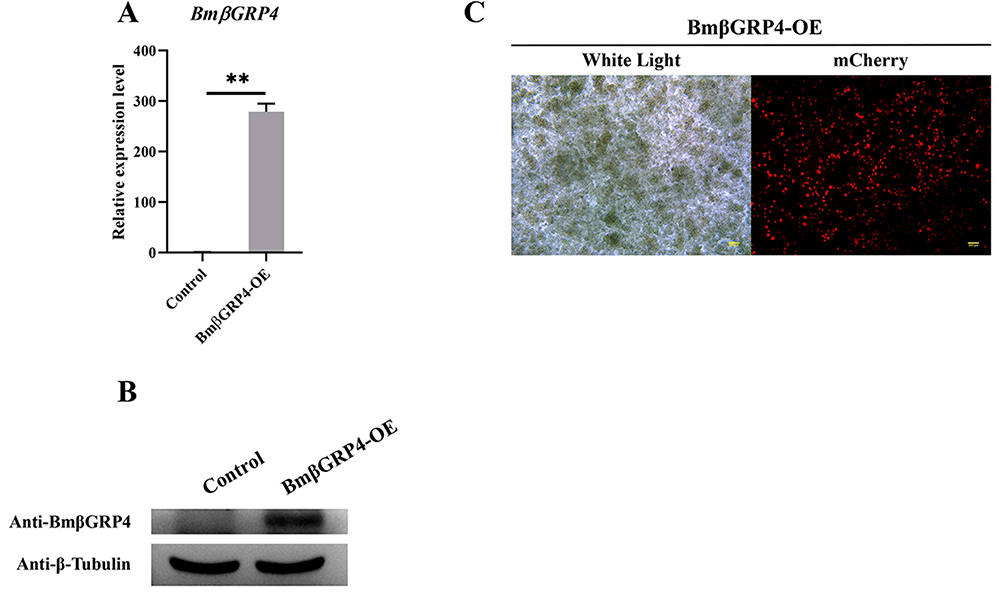
Overexpression of BmβGRP4 in BmN cells was confirmed by analyzing the transcript level (**A**), translational level (**B**) and observing the fluorescence signal (**C**) at 48 h after vector transfection using RT-qPCR, Western blot and an inverted fluorescence microscope (bar = 200 μm). White Light, optical transmission. mCherry, red fluorescence. Statistical analysis was conducted using SPSS software. Significant differences are indicated by ** (*p* < 0.01).

**Figure 7:**
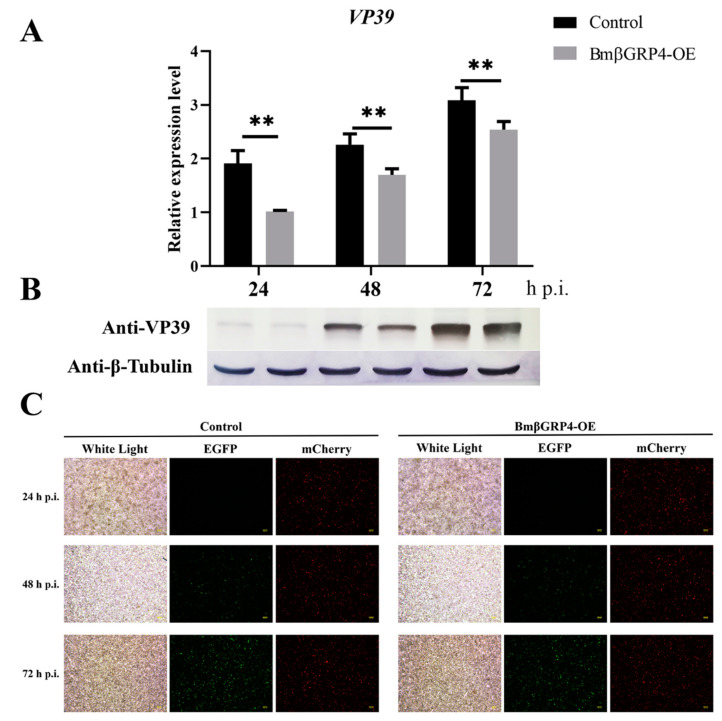
Overexpression of *BmβGRP4* suppressed the proliferation of the BV-EGFP in BmN cells. (**A**) RT-qPCR analysis of the relative expression levels of BmNPV *VP39* after overexpression of *BmβGRP4*. (**B**) Western blotting analysis of the translational levels of BmNPV VP39 after overexpression of *BmβGRP4*. (**C**) The infected cells (BmN cells with green fluorescence) were observed by an inverted fluorescence microscope at 24, 48 and 72 h p.i. (bar = 200 μm). White Light, optical transmission. EGFP, green fluorescence. mCherry, red fluorescence. Data were normalized using *BmGAPDH* and are presented as means ± SEM from three independent experiments. Statistical analysis was conducted using SPSS software. Significant differences are indicated by ** (*p* < 0.01).

To further study the role of *BmβGRP4* in BmNPV infection in vitro, a recombinant plasmid, pIZT-mCherry-BmβGRP4, was constructed to overexpress *BmβGRP4* in BmN cells. Subsequently, the recombinant plasmid was transfected into BmN cells. At 48 h after transfection, the red fluorescence signal was observed by fluorescence microscopy (Figure 6C); in addition, compared to the control group, the very significant upregulation of *BmβGRP4* was acquired in RT-qPCR analysis and Western blot analysis (Figure 6A,B). After infection with BV-EGFP, viral proliferation that was indicated by the green fluorescence signal was reduced significantly in BmβGRP4 overexpressed cells at 24, 48, and 72 h p.i. compared to that in the control group (Figure 7C). Furthermore, compared with the control group, viral loads represented by the expressions of *VP39* analyzed by RT-qPCR and Western blot were also remarkably decreased from 24 to 72 h p.i. (Figure 7A,B). Due to the very low expression of *BmβGRP4* in BmN cells, the knockdown of *BmβGRP4* could not be investigated. These results show that the overexpression of *BmβGRP4* could suppress BmNPV proliferation in BmN cells.

### 3.7. BmβGRP4 Promoted BmNPV Induced Apoptosis in BmN Cells

**Figure 8:**
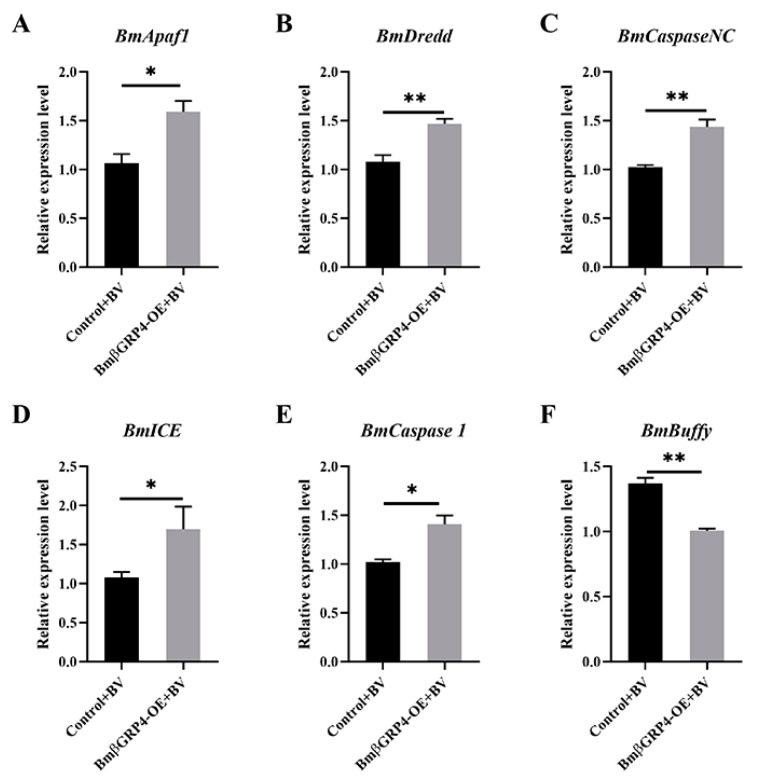
Expression analysis of selected apoptosis-related genes in *BmβGRP4* overexpressed BmN cells following BmNPV infection at 36 h p.i. The relative expression levels of *BmApaf1* (**A**), *BmDredd* (**B**), *BmCaspaseNC* (**C**), *BmICE* (**D**), *BmCaspase1* (**E**) and *BmBuffy* (**F**) through RT-qPCR. Data were normalized using *BmGAPDH* and are presented as means ± SEM from three independent experiments. Statistical analysis was conducted using SPSS software. Significant differences are indicated by * (*p* < 0.05) or ** (*p* < 0.01).

**Figure 9:**
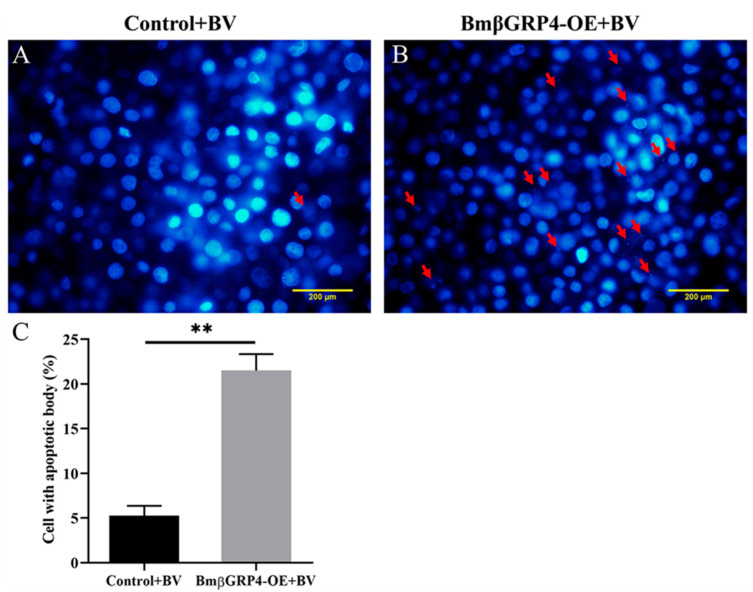
Overexpression of *BmβGRP4* promoted the formation of BmNPV-induced apoptotic bodies in BmN cells. (**A**,**B**) Fluorescence microscopic images of morphological changes in the nuclei of *BmβGRP4* overexpressed BmN cells stained with DAPI at 36 h p.i. The red arrows indicate apoptotic bodies. (**C**) Statistics of cells with apoptotic bodies. Statistical analysis was conducted using SPSS software with Student’s *t*-test. Significant differences are indicated by ** (*p* < 0.01).

Apoptosis plays a key role in the innate immunity of insects, serving as an important antiviral defense mechanism in insects [32,33]. To verify whether BmβGRP4 had the regulatory function in BmNPV induced apoptosis in BmN cells, we overexpressed BmβGRP4 by transfecting the recombinant pIZT-mCherry-BmβGRP4 vector in BmN cells, after which BV-EGFP was used to induce apoptosis. As shown in Figure 8, compared to the control, the relative expression levels of *BmApaf1*, *BmDredd*, *BmCaspaseNC*, *BmICE* and *BmCaspase1* were upregulated, and the relative expression level of *BmBuffy* was downregulated at 36 h p.i. In addition, cell apoptosis level was determined by DAPI staining. Numerous apoptotic bodies were observed in the infected cells at 36 h p.i. Over 20% of the *BmβGRP4* overexpressed cells had apoptotic bodies; however, only about 5% of the control cells had apoptotic bodies (Figure 9). Together, our results demonstrate that *BmβGRP4* promoted BmNPV induced apoptosis in BmN cells.

### 3.8. BmβGRP4 Positively Regulated BmPTEN and Negatively Regulated BmIAP

**Figure 10:**
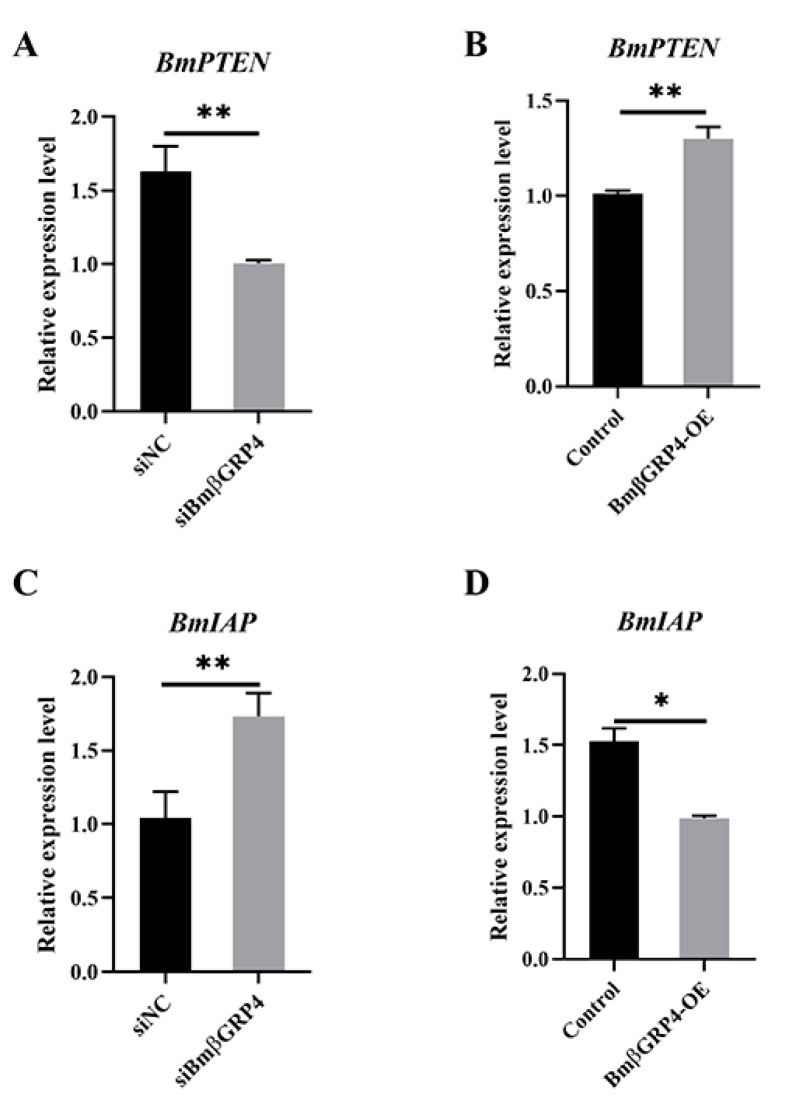
*BmβGRP4* positively regulates *BmPTEN* and negatively regulates *BmIAP*. The expression levels of *BmPTEN* after *BmβGRP4* knockdown in the midgut (**A**) and overexpression in BmN cells (**B**) were evaluated through RT-qPCR. The expression levels of *BmIAP* after *BmβGRP4* knockdown in the midgut (**C**) and the overexpression in BmN cells (**D**) were evaluated by RT-qPCR. Data were normalized using *BmGAPDH* and are presented as means ± SEM from three independent experiments. Statistical analysis was conducted using SPSS software with Student’s *t*-test. Significant differences are indicated by * (*p* < 0.05) or ** (*p* < 0.01).

According to the study of Jiang et al. [30], another important pattern recognition receptor BmPGRP2-2 could be induced in response to BmNPV infection, through PTEN-phosphoinositide 3-kinase (PI3K)/Akt signaling to regulate apoptosis. In addition, Chen et al. [31] have found that *B. mori* inhibitor of apoptosis (*BmIAP*) could inhibit apoptosis and increase BmNPV proliferation during BmNPV infection in silkworm cells. To further determine the possible way in which *BmβGRP4* regulates the response to BmNPV infection, we explored the effect of *BmβGRP4* on the expression of *BmPTEN* and *BmIAP*. The expression levels of *BmPTEN* and *BmIAP* were analyzed in the *BmβGRP4* knockdown midgut and *BmβGRP4* overexpressed BmN cells using RT-qPCR. As shown in Figure 10, the expression level of *BmPTEN* was significantly decreased when *BmβGRP4* was knocked down in the midgut, while *BmβGRP4* overexpression in BmN cells significantly increased the expression of *BmPTEN*. Conversely, the expression level of *BmIAP* was significantly increased when *BmβGRP4* was knocked down, and significantly decreased when *BmβGRP4* was overexpressed. These results indicate that *BmPTEN* is positively regulated by *BmβGRP4*, whereas *BmIAP* is negatively regulated by *BmβGRP4*.

## 4. Discussion

BmNPV is a major pathogen that specifically infects silkworms and causes serious loss in sericulture every year. The silkworm midgut is an important barrier against BmNPV infection [8]. Recently, many proteins related to BmNPV infection have been reported. Previously, a *B. mori* β-1,3-glucan recognition protein named *BmβGRP4* was identified from a transcriptome database [7]. β-1,3-glucan recognition proteins (βGRPs) are known to be pattern recognition molecules that play an important role in innate immunity activation and regulation [17]. In this study, we demonstrated that *BmβGRP4* showed a very significant downregulation in susceptible strain P50 after BmNPV infection (Table S1); hence, we speculated that *BmβGRP4* might be related to BmNPV infection. The bioinformatic analysis results show that the *BmβGRP4* amino acid sequence contained the typical βGRP domains (Glycoside hydrolase family 16) and seven predicted glycosylation sites (Figure 1). Glycoside hydrolase is a widespread group of enzymes that hydrolyze the glycosidic bond between two or more carbohydrates. In other insects, *M. sexta* βGRP2 and *Ostrinia furnacalis* βGRP3 also have a conserved glycoside hydrolase family 16 domain [17]. A phylogenetic tree was generated from the analysis of amino acid sequences of the βGRPs from other insects to verify the evolutionary relationship of βGRPs; it indicated that BmβGRP4 shares a close relationship with *P. xylostella* βGRP. The RT-qPCR analysis showed that BmβGRP4 was highly expressed in the 1st to 5th instar larval stages and the expression level increased with instar stage. Additionally, it was expressed in almost all of the selected tissues, and was highly expressed in the head, body fat and hemocyte, especially in the midgut (Figure 2A). During the larval stage, silkworms had to eat a large number of mulberry leaves, which also meant that they would face a large number of microbial infections. For this reason, silkworms had to improve the expression of some immune-related genes to adapt to the living environment. In addition, larvae of lepidopteran insects commonly become increasingly resistant to virus infections as they age [34]. βGRPs are known as pattern recognition molecules that play an important role in innate immune response. BmβGRP4 might also be related to the innate immune response; hence, we speculated that the increased expression of BmβGRP4 with instar stage would be beneficial to the enhancement of immunity in silkworm larvae. Previous studies found that GRPs in *M. sexta* [35], *P. interpunctella* [36] and *O. furnacalis* [37] were mainly transcribed in the fat body and hemocyte. This indicated that *BmβGRP4* might possess different functions compared to those reported in other insects. 

Due to the significant decrease in *BmβGRP4* transcription found from the data of the transcriptome, we consider *BmβGRP4* to play an important role in BmNPV infection. The RT-qPCR analysis showed that the expression of *BmβGRP4* was inhibited in the P50 strain midgut after BmNPV infection (Figure 4A), and the Western blot analysis also showed a similar result (Figure 4B). We speculated that BmβGRP4 might be involved in resistance to BmNPV replication in the midgut. To verify the speculation, the expression of BmNPV *VP39* was detected in viral-infected knockdown of *BmβGRP4* in larvae and *BmβGRP4* overexpressed in BmN cells. A significant increase in replication was shown after the knockdown of *BmβGRP4* in the 5th instar larvae midgut (Figure 5), while the reduced proliferation of BmNPV was observed after the overexpression of *BmβGRP4* in BmN cells (Figure 6). Therefore, these results further confirm that *BmβGRP4* was related to BmNPV infection. Previously, several proteins identified from the digestive juice of *B. mori* larvae showed that the overexpression of these proteins would significantly reduce the proliferation of BmNPV in BmN cells [29,38,39]. We consider *BmβGRP4* to have an antiviral function.

**Figure 11:**
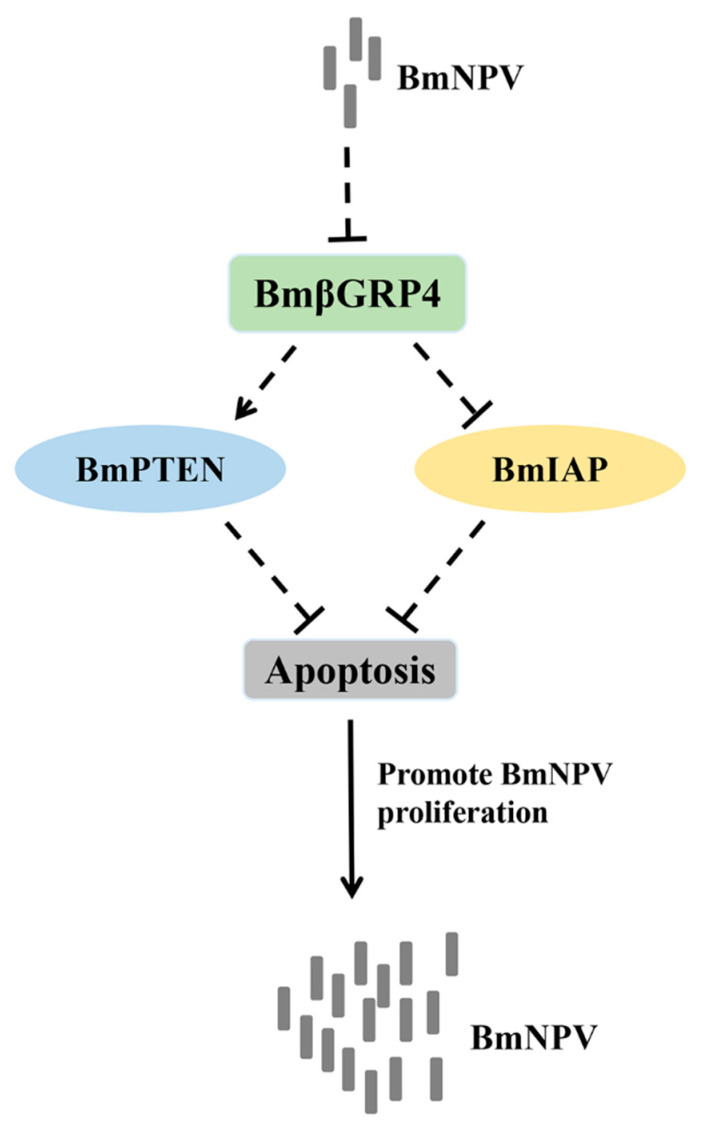
The schematic diagram shows the putative mechanism of *BmβGRP4* in BmNPV infection. *BmβGRP4* is suppressed by BmNPV to suppress *BmPTEN* and induce *BmIAP*, thereby inhibiting cell apoptosis. The increased cell survival is beneficial for BmNPV replication.

Apoptosis plays a key role in the innate immunity of insects, serving as an important antiviral defense mechanism in insects [32,33,40]. To verify whether *BmβGRP4* had a regulatory function in BmNPV induced apoptosis, we overexpressed *BmβGRP4* in BmN cells and then BV-EGFP was used to induce apoptosis. As shown in Figure 8, compared to the control, the relative expression levels of several apoptosis-related genes *BmApaf1*, *BmDredd*, *BmCaspaseNC*, *BmICE* and *BmCaspase1* were upregulated, while the relative expression level of *BmBuffy* was downregulated at 36 h p.i. In addition, cell apoptosis level was determined by DAPI staining, and more apoptotic bodies were observed in the cells *BmβGRP4* overexpressed at 36 h p.i. (Figure 9). These results demonstrate that *BmβGRP4* promoted BmNPV-induced apoptosis in BmN cells. According to the study of Jiang et al. [30], another important pattern recognition receptor BmPGRP2-2 could be induced to regulate apoptosis in response to BmNPV infection, through PTEN-phosphoinositide 3-kinase (PI3K)/Akt signaling. In addition, Chen et al. [31] found that the *B. mori* inhibitor of apoptosis (*BmIAP*) could inhibit apoptosis and increase BmNPV proliferation during BmNPV infection in silkworm cells. We found that *BmβGRP4* positively regulated *BmPTEN* through the PTEN-PI3K/Akt signaling to regulate cell apoptosis and *BmβGRP4* negatively regulated *BmIAP* to regulate cell apoptosis (Figure 10). Hence, we hypothesize that *BmβGRP4* was inhibited by BmNPV infection to suppress the expression of *BmPTEN* and facilitate the expression of *BmIAP* to inhibit apoptosis to promote viral replication (Figure 11). The mechanism underlying the positive regulation of *BmPTEN* and negative regulation of *BmIAP* by *BmβGRP4* still needs further research. Furthermore, it is still unclear whether BmβGRP4 induces apoptosis by itself or requires a pathogen to be present, and how it works.

## 5. Conclusions

In summary, a differentially expressed β-1,3-glucan recognition protein, *BmβGRP4*, was identified from a transcriptome database. The significant downregulation of *BmβGRP4* expression was detected after BmNPV infection in the P50 larvae midgut. Subsequently, the overexpression of *BmβGRP4* suppressed BmNPV proliferation in BmN cells; however, the siRNA-mediated knockdown of *BmβGRP4* facilitated BmNPV proliferation in *B. mori* larvae. Furthermore, we demonstrated that *BmβGRP4* overexpression promoted BmNPV-induced cellular apoptosis. Then, we found that *BmβGRP4* positively regulated *BmPTEN* and negatively regulated *BmIAP*. Consequently, we speculated that BmNPV inhibited *BmβGRP4* to suppress *BmPTEN* and facilitate *BmIAP* to inhibit cell apoptosis to evade the host antiviral defense. The findings of this study will lay a foundation for further study of the functions of *BmβGRP4* in response to BmNPV.

## Data Availability

The data presented in this study are contained within the article and Supplementary Materials.

## Supplementary Materials

The following are available online at https://www.mdpi.com/article/10.3390/insects12080743/s1, Table S1: The transcriptome data statistics of DEG *BmβGRP4*, Table S2: The primers used in this study, Table S3: The siRNA sequences used in this study.

## References

[1] Xia Q., Zhou Z., Lu C., Cheng D., Dai F., Li B., Zhao P., Zha X., Cheng T., Chai C. (2005). A Draft Sequence for the Genome of the Domesticated Silkworm (Bombyx Mori). Science.

[2] Kato T., Kajikawa M., Maenaka K., Park E.Y. (2010). Silkworm Expression System as a Platform Technology in Life Science. Appl. Microbiol. Biotechnol..

[3] The International Silkworm Genome Consortium (2008). The Genome of a Lepidopteran Model Insect, the Silkworm Bombyx Mori. Insect Biochem. Mol. Biol..

[4] Hu X., Shen Y., Zheng Q., Wang G., Wu X., Gong C. (2016). Bm59 Is an Early Gene, but Is Unessential for the Propagation and Assembly of Bombyx Mori Nucleopolyhedrovirus. Mol. Genet. Genom..

[5] Ponnuvel K.M., Nakazawa H., Furukawa S., Asaoka A., Ishibashi J., Tanaka H., Yamakawa M. (2003). A Lipase Isolated from the Silkworm Bombyx Mori Shows Antiviral Activity against Nucleopolyhedrovirus. J. Virol..

[6] Cheng Y., Wang X.-Y., Du C., Gao J., Xu J.-P. (2014). Expression Analysis of Several Antiviral Related Genes to Bmnpv in Different Resistant Strains of Silkworm, Bombyx Mori. J. Insect Sci..

[7] Wang X.-Y., Yu H.-Z., Geng L., Xu J.-P., Yu D., Zhang S.-Z., Ma Y., Fei D.-Q. (2016). Comparative Transcriptome Analysis of Bombyx Mori (Lepidoptera) Larval Midgut Response to Bmnpv in Susceptible and near-Isogenic Resistant Strains. PLoS ONE.

[8] Yu H.-Z., Wang X.-Y., Xu J.-P., Ma Y., Zhang S.-Z., Yu D., Fei D.-Q., Muhammad A. (2017). Itraq-Based Quantitative Proteomics Analysis of Molecular Mechanisms Associated with Bombyx Mori (Lepidoptera) Larval Midgut Response to Bmnpv in Susceptible and near-Isogenic Strains. J. Proteom..

[9] Xue J., Qiao N., Zhang W., Cheng R.L., Zhang X.Q., Bao Y.Y., Xu Y.P., Gu L.Z., Han J.D., Zhang C.X. (2012). Dynamic Interactions between Bombyx Mori Nucleopolyhedrovirus and Its Host Cells Revealed by Transcriptome Analysis. J. Virol..

[10] Dong X.L., Liu T.H., Wang W., Pan C.X., Wu Y.F., Du G.Y., Chen P., Lu C., Pan M.H. (2015). Bmreepa Is a Novel Gene That Facilitates Bmnpv Entry into Silkworm Cells. PLoS ONE.

[11] Kawai T., Akira S. (2007). Antiviral Signaling through Pattern Recognition Receptors. J. Biochem..

[12] Chen Y.Y., Chen J.C., Lin Y.C., Kitikiew S., Li H.F., Bai J.C., Tseng K.C., Lin B.W., Liu P.C., Shi Y.Z. (2014). Endogenous Molecules Induced by a Pathogen-Associated Molecular Pattern (Pamp) Elicit Innate Immunity in Shrimp. PLoS ONE.

[13] Lu Y., Su F., Li Q., Zhang J., Li Y., Tang T., Hu Q., Yu X.-Q. (2020). Pattern Recognition Receptors in Drosophila Immune Responses. Dev. Comp. Immunol..

[14] Gobert V., Gottar M., Matskevich A.A., Rutschmann S., Royet J., Belvin M., Hoffmann J.A., Ferrandon D. (2003). Dual Activation of the Drosophila Toll Pathway by Two Pattern Recognition Receptors. Science.

[15] Zhang X., He Y., Cao X., Gunaratna R.T., Chen Y.R., Blissard G., Kanost M.R., Jiang H. (2015). Phylogenetic Analysis and Expression Profiling of the Pattern Recognition Receptors: Insights into Molecular Recognition of Invading Pathogens in Manduca Sexta. Insect Biochem. Mol. Biol..

[16] Anjugam M., Vaseeharan B., Iswarya A., Amala M., Govindarajan M., Alharbi N.S., Kadaikunnan S., Khaled J.M., Benelli G. (2017). A Study on Β-Glucan Binding Protein (Β-Gbp) and Its Involvement in Phenoloxidase Cascade in Indian White Shrimp Fenneropenaeus Indicus. Mol. Immunol..

[17] Jiang H., Ma C., Lu Z.-Q., Kanost M.R. (2004). Beta-1,3-Glucan Recognition Protein-2 (Betagrp-2) from Manduca Sexta; an Acute-Phase Protein That Binds Beta-1,3-Glucan and Lipoteichoic Acid to Aggregate Fungi and Bacteria and Stimulate Prophenoloxidase Activation. Insect Biochem. Mol. Biol..

[18] Steiner H. (2004). Peptidoglycan Recognition Proteins: On and Off Switches for Innate Immunity. Immunol. Rev..

[19] Krem M.M., Cera E.D. (2002). Evolution of Enzyme Cascades from Embryonic Development to Blood Coagulation. Trends Biochem. Sci..

[20] Nappi A.J., Vass E. (2001). Cytotoxic Reactions Associated with Insect Immunity. Adv. Exp. Med. Biol..

[21] Ochiai M., Ashida M. (1988). Purification of a Beta-1,3-Glucan Recognition Protein in the Prophenoloxidase Activating System from Hemolymph of the Silkworm, Bombyx Mori. J. Biol. Chem..

[22] Huang W., Xu X., Freed S., Zheng Z., Shuang W., Ren S., Jin F. (2015). Molecular Cloning and Characterization of a Β-1,3-Glucan Recognition Protein from Plutella Xylostella (L.). New Biotechnol..

[23] Thompson M.R., Kaminski J.J., Kurt-Jones E.A., Fitzgerald K.A. (2011). Pattern Recognition Receptors and the Innate Immune Response to Viral Infection. Viruses.

[24] Kawai T., Akira S. (2006). Innate Immune Recognition of Viral Infection. Nat. Immunol..

[25] Heiberg I.L., Winther T.N., Paludan S.R., Hogh B. (2012). Pattern Recognition Receptor Responses in Children with Chronic Hepatitis B Virus Infection. J. Clin. Virol..

[26] Tsai C.W., McGraw E.A., Ammar E.D., Dietzgen R.G., Hogenhout S.A. (2008). Drosophila Melanogaster Mounts a Unique Immune Response to the Rhabdovirus Sigma Virus. Appl. Environ. Microbiol..

[27] Gao K., Deng X.-Y., Qian H.-Y., Qin G.-X., Hou C.-X., Guo X.-J. (2014). Cloning and Expression Analysis of a Peptidoglycan Recognition Protein in Silkworm Related to Virus Infection. Gene.

[28] Tamura K., Peterson D., Peterson N., Stecher G., Nei M., Kumar S. (2011). Mega5: Molecular Evolutionary Genetics Analysis Using Maximum Likelihood, Evolutionary Distance, and Maximum Parsimony Methods. Mol. Biol. Evol..

[29] Zhang S.-Z., Zhu L.-B., You L.-L., Wang J., Cao H.-H., Liu Y.-X., Toufeeq S., Wang Y.-L., Kong X., Xu J.-P. (2020). A Novel Digestive Proteinase Lipase Member H-a in Bombyx Mori Contributes to Digestive Juice Antiviral Activity against B. Mori Nucleopolyhedrovirus. Insects.

[30] Jiang L., Liu W., Guo H., Dang Y., Cheng T., Yang W., Sun Q., Wang B., Wang Y., Xie E. (2019). Distinct Functions of Bombyx Mori Peptidoglycan Recognition Protein 2 in Immune Responses to Bacteria and Viruses. Front. Immunol..

[31] Chen P., Kang T.-T., Bao X.-Y., Dong Z.-Q., Zhu Y., Xiao W.-F., Pan M.-H., Lu C. (2020). Evolutionary and Functional Analyses of the Interaction between the Bombyx Mori Inhibitor of Apoptosis (Iap) and Nucleopolyhedrovirus Iaps. Insect Sci..

[32] Everett H., McFadden G. (1999). Apoptosis: An Innate Immune Response to Virus Infection. Trends Microbiol..

[33] Clarke T.E., Clem R.J. (2003). Insect Defenses against Virus Infection: The Role of Apoptosis. Int. Rev. Immunol..

[34] Engelhard E.K., Volkman L.E. (1995). Developmental Resistance in Fourth Instar Trichoplusia Ni Orally Inoculated with Autographa Californica M Nuclear Polyhedrosis Virus. Virology.

[35] Ma C., Kanost M.R. (2000). A Β1,3-Glucan Recognition Protein from an Insect, Manduca Sexta, Agglutinates Microorganisms and Activates the Phenoloxidase Cascade. J. Biol. Chem..

[36] Fabrick J.A., Baker J.E., Kanost M.R. (2003). Cdna Cloning, Purification, Properties, and Function of a Beta-1,3-Glucan Recognition Protein from a Pyralid Moth, Plodia Interpunctella. Insect Biochem. Mol. Biol..

[37] Wu T., Zhao Y., Wang Z., Song Q., Wang Z., Xu Q., Wang Y., Wang L., Zhang Y., Feng C. (2017). Β-1,3-Glucan Recognition Protein 3 Activates the Prophenoloxidase System in Response to Bacterial Infection in Ostrinia Furnacalis Guenée. Dev. Comp. Immunol..

[38] Li G., Zhou Q., Qiu L., Yao Q., Chen K., Tang Q., Hu Z. (2017). Serine Protease Bm-Sp142 Was Differentially Expressed in Resistant and Susceptible Bombyx Mori Strains, Involving in the Defence Response to Viral Infection. PLoS ONE.

[39] Cao H.-H., Zhang S.-Z., Zhu L.-B., Wang J., Liu Y.-X., Wang Y.-L., Kong X., You L.-L., Toufeeq S., Liu S.-H. (2021). The Digestive Proteinase Trypsin, Alkaline a Contributes to Anti-Bmnpv Activity in Silkworm (Bombyx Mori). Dev. Comp. Immunol..

[40] Marques J.T., Imler J.-L. (2016). The Diversity of Insect Antiviral Immunity: Insights from Viruses. Curr. Opin. Microbiol..

