# Supplementary material for: Bombyx mori β-1,3-Glucan Recognition Protein 4 (BmβGRP4) Could Inhibit the Proliferation of B. mori Nucleopolyhedrovirus through Promoting Apoptosis
Source: Insects. 2021 Aug 18;12(8):743. doi: 10.3390/insects12080743 (PMC8396850; doi:10.3390/insects12080743)
Supplement: Supplementary file 1 [file insects-12-00743-s001.zip › Supplementary materials.pdf]

Table S1. The transcriptome data statistics of DEG *BmβGRP4*.

Gene ID	Gene Description	Accession No.	P50+/P50-
BGIBMGA000353	beta-1,3-glucan recognition protein 4 precursor [*Bombyx mori*]	NM_001166142.1	0.014

Table S2. The primers used in this study.

Primer name	Sequence (5′-3′)	Purpose
BmβGRP4-F	CCGGAATTC*TGCACACCCAGCGTAAC (*EcoR I*)	Protein expression
BmβGRP4-R	CCGCTCGAGTTATAACGCCCAAACTCG (*Xho I*)	
OEBmβGRP4-F	CCGGAATTCATGTGGCTGTTAACTCTGGG (*EcoR I*)	Overexpression
OEBmβGRP4-R	CCGCTCGAGTAACGCCCAAACTCGAAC (*Xho I*)	
qBmβGRP4-F	ACCCAGCGTAACGACAGTGAG	RT-qPCR
qBmβGRP4-R	TAAAGTGTTTTCGTGCTGCCAT	
BmGAPDH-F	CATTCCGCGTCCCTGTTGCTAAT	
BmGAPDH-R	GCTGCCTCCTTGACCTTTTGC	
VP39-F	CAACTTTTTGCGAAACGACTT	
VP39-R	GGCTACACCTCCACTTGCTT	
qBmApaf1-F	ACAGTTCACAACCCTCTAAAATCAC	
qBmApaf1-R	CACTTTCTTACCACGCATCACC	
qBmDredd-F	TAATAGTCGTTCTGACTTGGGACA	
qBmDredd-R	TCGGTATGCAATGCAGTTTCT	
qBmCaspaseNC-F	TTCCCAGCAAATGATAGAACCA	
qBmCaspaseNC-R	ATCAAGGTCACGCACCAAATC	
qBmICE-F	CCTCATCCATAAAGGGACCAC	
qBmICE-R	CTGCCGACCAACCATACAAG	
qBmCaspase1-F	AAGAGCCACGAGTTGTGTTT	
qBmCaspase1-R	TGCCAAGGTGATAAGTTAGA	
qBmBuffy-F	TCAGCTATGCTACGCTCAGACA	
qBmBuffy-R	ATCCATGATCCAGGCTCCTC	
qBmPTEN-F	CTGATAGTGGAGAAGGTGCCG	
qBmPTEN-R	GTAATGGCCGACGCGCT	
qBmIAP-F	TGACGAAAGTTGCTAAAAATGGA	
qBmIAP-R	GGTGACGGGAGTGTGGATGT	

* The underline represented restriction enzyme cutting site.

Table S3. The siRNA sequences used in this study.

siRNA name	Sequence (5′-3′)	Length (bp)
siBmβGRP4-Sense	GCCCUAAUAUCGGCGAGUATT	21
siBmβGRP4-Anti-sense	UACUCGCCGAUAUUAGGGCTT	21
siNC-Sense	UUCUCCGAACGUGUCACGUTT	21
siNC-Anti-sense	ACGUGACACGUUCGGAGAATT	21
